# Ovarian pedicle hemostasis techniques in cats

**DOI:** 10.1590/ACB360308

**Published:** 2021-05-07

**Authors:** Maylane Tavares Ferreira da Silva, Alex Cardoso de Melo, Cristiano Francisco Barros do Nascimento, Filipi Alexandre do Nascimento Silva, Talita Banchin Borges, Ana Maria Quessada, Marcelo Campos Rodrigues

**Affiliations:** 1 Graduate student. Universidade Federal do Piauí – Center of Agricultural Sciences – Department of Clinical and Veterinary Surgery – Teresina (PI), Brazil.; 2 Resident. Universidade Federal do Piauí – Center of Agricultural Sciences – Veterinary Hospital Jeremias Pereira da Silva – Teresina (PI), Brazil.; 3 Veterinarian. Universidade Federal do Piauí – Center of Agricultural Sciences – Veterinary Hospital Jeremias Pereira da Silva – Universidade Federal do Piauí – Teresina (PI), Brazil.; 4 Fellow PhD degree. Universidade Federal do Piauí – Postgraduate Program in Animal Science – Teresina (PI), Brazil.; 5 Fellow PhD degree. Universidade Paranaense – Postgraduate Program in Animal Science – Umuarama (PR), Brazil.; 6 Full Professor. Universidade Paranaense – Postgraduate Program in Animal Science – Umuarama (PR), Brazil.; 7 Full Professor. Universidade Federal do Piauí – Department of Clinical and Veterinary Surgery – Veterinary Surgical Clinic – Teresina (PI), Brazil.

**Keywords:** Castration, Ovariohysterectomy, Surgical, Feline

## Abstract

**Purpose:**

To evaluate hemostasis of the ovarian arteriovenous complex (OAVC) in relation to surgical time, practicality and feasibility in three ovariohysterectomy (OH) techniques for queens.

**Methods:**

The experiment was performed on 21 female cats aged between six months and seven years, randomly arranged into three groups in a completely randomized design. Group one was spayed using the conventional three-clamp technique, group two using the OAVC knotting technique, and group three using the ovarian pedicle rotation technique. The student’s t-test and Tukey’s test were used to compare the mean surgical times.

**Results:**

The conventional technique, which uses thread wires, was more laborious and required longer execution time compared to the other two techniques. The OAVC knotting technique was the fastest and had the least blood loss.

**Conclusions:**

The use of techniques that do not use synthetic materials for OAVC hemostasis was proven to be appropriate in castration projects, provided that the surgical team has sufficient training.

## Introduction

Several techniques have been used to ligate the pedicles and uterine body of cats in ovariohysterectomy (OH)^1^–^4^. However, postoperative complications may occur in all techniques with hemorrhage as one of the most common complications and the most common cause of death in postoperative OH; this can be caused by ruptured ovarian and uterine vessels during resection^5^. Thus, the hemostasis of these vessels is important in OH.

The obliteration of the ovarian arteriovenous complex (OAVC) in cats can be performed by rotating the OAVC around its own axis after incision of the ovarian veins and arteries^3^ and by a maneuver in which a knot is made on its own axis^2^. These techniques need no thread or other synthetic materials for OAVC hemostasis.

Few studies are found in the literature addressing the topic. The objective of this study was to compare the OH techniques that use of surgical thread wires with the conventional three-clamp technique.

## Methods

This study was approved by the Ethics Committee on the Use of Animals of the Universidade Federal do Piauí (UFPI) with registration No. 582/19.

A total of 21 healthy female cats aged between six months and seven years were spayed. Only clinically healthy cats were selected, regardless of breed and body mass.

The queens were randomly distributed into three groups of seven. The female cats in the first group (CG) were operated using common suture thread (polyglactin 910) to perform OAVC ligature, this is the conventional technique^1^,^4^. The second group (KG) were operated using the knotting technique, in which the ovarian vessels were occluded by knotting the OAVC itself^2^. In the third group (RG), the OAVC was resected and rotated in its own axis to occlude it^3^.

### Anesthesia and surgical procedures

The surgical procedures were performed by the same surgical team. Surgical time was recorded from the beginning of laparotomy to the end of dermorraphy. The hematocrit was measured 24 h before and 48 h after surgery.

In the preoperative period, after hydric and food fasting for three and 12 h, respectively, sodium cephalothin(25 mg/kg) and meloxicam (0.2 mg/kg) were administered. Such drugs were administered intramuscularly (IM) 20 min before preanesthetic medication (MPA).

Cephalic vein venipuncture for fluid therapy with 0.9% sodium chloride solution (5 mL/kg/h) was performed. Epidural anesthesia was performed at the lumbosacral junction with 2% lidocaine with vasoconstrictor (0.25 mL/kg). The animals were submitted to MPA and induction with acepromazine (0.03 mg/kg), morphine (0.2 mg/kg), midazolam (0.12 mg/kg)and ketamine (8 mg/kg). These drugs were administered in the same syringe intramuscularly. Ten minutes after this medication, the cats were intubated with an orotracheal tube and anesthetic maintenance was performed with isoflurane.

**Figure 1:**
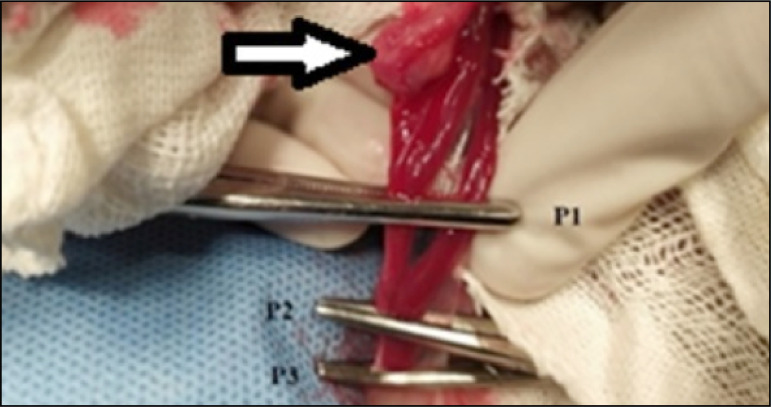
Three-clamp technique for ovariectomy in a cat. Arrow: ovary; P1: clamp 1, caudal to the ovary; P2: clamp 2, caudal to P1; P3: clamp 3, caudal to P2. The pedicle section will be performed between P1 and P2.

After opening the abdominal cavity using an OH hook, the right ovary was exteriorized, the ovarian suspension ligament, ovarian bursa and OAVC were identified, and the ovarian suspension ligament was resected. In CG, a fenestra in the broad ligament was made immediately caudal to the OAVC. The ovarian pedicle was clamped with three Halsted hemostatic clamps (Fig. 1) and resected between the first and second ventral clamps (P1 and P2). The ovarian pedicle was ligated dorsally to the third forceps (P3) using 3-0 polyglactin 910, which was removed as the ligature was tightened. The pedicle was held between the thumb and forefinger, and the second forceps (P2) was released. After verifying that there was no hemorrhage in the pedicle, the stump was directed to its anatomical position. The same procedure was performed on the contralateral ovary.

**Figure 2:**
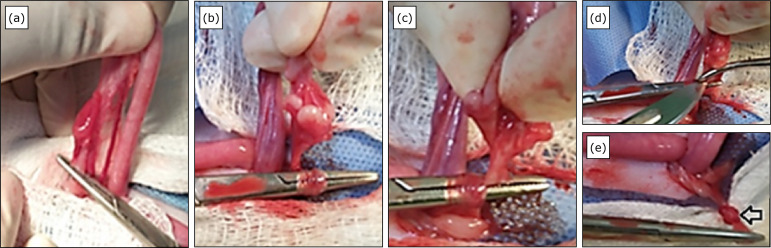
Knot-making technique with rotation on own the axis of the ovarian arteriovenous complex of the cat’s ovarian pedicle. **(a)** Clamping of the suspensory ligament for its section; **(b)** Rotation of the OAVC on its own axis to obtain the node; **(c)** After the movement, clamping the OAVC with the tip of the clamp to finish making the knot; **(d)** Section of the pedicle; **(e)** Finished node (arrow).

In the KG, the pedicle was rotated 360° on its own axis (Fig. 2b) in a medial to lateral direction using the Halstead curved hemostatic forceps at an angle of 45°. The tip of the forceps was fixed transversally to the proximal endof the pedicle. The second hemostatic forceps was placed proximal to the ovary and transversally to the pedicle. The OAVC was resected between P1 and P2, and a knot was formed by rotating the pedicle in its own axis; it was finished using a sterile bandage gauze (Fig. 2e). After verifying that there was no hemorrhage, the same procedure was performed on the contralateral ovary.

**Figure 3:**
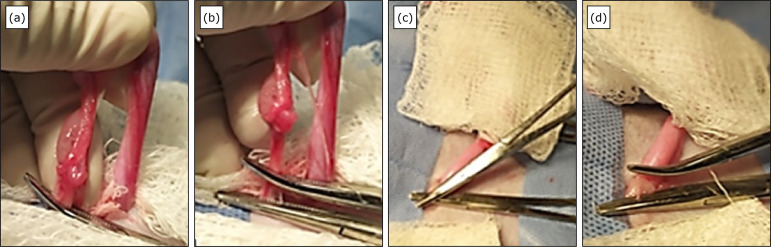
Rotation technique on the ovarian arteriovenous complex performed in the ovarian pedicle of a cat. **(a)** Placement of curved hemostatic forceps transversally to the OAVC; **(b)** Straight hemostatic forceps placed ventral to the curved forceps; **(c)** Moment after the ovarian pedicle resection and before the rotation maneuver; **(d)** Aspect of the rotated stump.

In RG, after resecting the suspensory ligaments of ovary and broad ligament of uterus on the right antimere, three Halstead forceps were placed on the OAVC, which was resected between the two ventral-most clamps(Fig. 3). The remaining ventral forceps were rotated until there was resistance; this was achieved with a mean of eight clockwise rotations, while the dorsal forceps remained fixed. Then, the ventral forceps were released, followed by the dorsal forceps. After verifying that there was no hemorrhage, the same procedure was performed on the contralateral OAVC.

After the procedures performed on the pedicles, the broad ligament was released bilaterally. The uterine body was ligated in the three groups according to the technique recommended in the literature^1^. The abdominal wall was sutured with 2-0 monofilament nylon thread in a separate pattern, and the subcutaneous layer was sutured using 3-0 polyglactin 910 thread in a continuous pattern. The skin was sutured using 2-0 monofilament nylon in a separate Wolf pattern.

### Postoperative

After surgery, all animals in this study were observed for 48 h to monitor the surgical wound, assess pain, and perform abdominal ultrasonography and postsurgical hematocrit test 48 h after.

### Statistical analysis

Analysis of variance was performed. Tukey’s test (p < 0.05)was used to compare the means of the three groups whenthere was a significance in F test (p < 0.05). The use ofseven repetitions per group provided 21 degrees of freedom for the residue. As for hematocrit analysis, the t-test was used on the means of two samples with 2n-n degrees of freedom.

## Results

The animals were classified as ASA I (normal healthy subjects)^6^ based on clinical examination (including hematocrit). None of the cats that had undergone the surgical procedure died.

No hemorrhages and loss of pedicles occurred during the operations. All cats presented good postoperative recovery. There were no postoperative infections, dehiscence, or any other intercurrences.

An abdominal ultrasound performed 48 h after surgery showed no pathological changes, or presence of free fluid in the abdominal cavity.

In the analysis of surgical times, KG was faster compared to CG and RG. The Tukey’s test for comparison of means showed a significant surgical time difference between CG (25.8 ± 5.9 min) and KG (16.96 ± 3.4 min), and there was no significant difference between CG and RG (19.43 ± 3.5 min). There was also no significant difference between the means of KG and RG.In the hematocrit analysis 48h after surgery, there was statistical difference between the CG and the other groups (Table 1).

The comparison of surgical times between the techniques used showed that KG had a significantly shorter surgical time compared to CG, which was statistically significant. The RG was performed faster than CG.The level of difficulty of RG was higher than KG.There was no difficulty in exposing the ovarian pedicles. However, OAVC hemostasis was easier observed in KG and RG compared to CG.

## Discussion

The absence of deaths among the female cats is probably related to the fact that healthy animals were classified as ASA I. Among animals classified as ASA I, death is not common^6^. In studies on the same topic, there were also no deaths^1^,^4^.

In the hematocrit analysis 48 h after surgery, the statistical difference between the CG and the groups (Table 1) may be related to the loss of blood volume accumulated between the P1 and P2 (Fig. 1), which was released after resection. In cases of acute bleeding, the immediate blood loss results in little to no change in hematocrit due to simultaneous loss of red blood cells and plasma fluid. During the following hours, the plasma volume was restored by extravascular space fluid, the hematocrit decreased^7^. However, no cats presented free fluid in the abdominal cavity on ultrasonographic evaluation, showing no postoperative hemorrhage. There were also no trans operative complications. In studies of OH in cats, the results were similar with no bleeding and trans operative complications^1^,^4^.

**Table 1:** Comparison of ovariohysterectomy techniques in cats (three groups, n = 21). Descriptive and comparative analysis of hematocrit values 24 h before and 48 h after surgery between groups.

Hematocrit	Groups	Mean (%)	Median (%)	Standard deviation	T_TAB_	RL (5%)	T_CAL_
HT1	CG	37.00	37.0	2.13	1.62	35.04–38.95	2.17
	KG	35.42	33.0	4.92		30.89–39.95	
	CG	37.00	37.0	2.13	2.17	35.04–38.95	0.17
	RG	36.85	38.0	3.48		33.66–40.04	
	KG	35.42	33.0	4.92	2.17	30.89–39.95	-1.34
	RG	36.85	38.0	3.48		33.66–40.04	
HT2	CG	30.85	33.0	2.64	2.17	28.41–33.29	-3.33
	KG	34.42	36.0	5.92		28.96–39.88	
	CG	30.85	33.0	2.64	2.17	28.41–33.29	-3.65
	RG	33.85	36.0	2.41		31.63–36.07	
	KG	34.42	36.0	5.92	2.17	28.96–39.88	0.54
	RG	33.85	36.0	2.41		31.63–36.07	

HT1 - hematocrit 24 h before surgery; HT2 - hematocrit 48 h after surgery; CG - conventional technique; KG - knotting technique; RG - rotation technique; RL (5%) - reliability limit; T_CAL_ - calculated T value; T_TAB_ - tabulated T value.

The comparison of surgical times between the techniques used showed that KG had a statistically significant shorter surgical time compared to CG. However, another study comparing the two techniques in cats showed no statistical difference^2^. These variations may be related to the number of experimental animals, since, in their study, each group had 11 female cats. In the study, each group was composed of seven queens.

A study comparing CG with KG reported that the degree of ingurgitation and the caliber of the vessel were relevant factors in performing the knot. In this case, the main difficulty was during the traction of the knot loopon the forceps, because with the ingurgitation of the vessel, the maneuver became more difficult^2^. This was minimized in the present study with the use of sterile gauze during traction of the knot loop on the forceps.

In the same article already cited and that is similar to present study, comparing CG with KG, the former was faster than the latter technique^2^. However, in this study, KG was performed faster than the others. In practice, there was a tendency to perform the described technique faster than the conventional technique. This is probably related to the learning curve^8^. In addition, the nonuse of sutures in pedicles optimized the surgical time^4^,^9^.

The literature describes that the ligature performed with pedicle knot around its own axis requires attention in rotating the hemostatic forceps on the pedicle, keeping it inclined at an angle of approximately 45°. When performed incorrectly, the maneuver becomes difficult to perform and weakens the tissue^2^. Such observations were also noticed in the present study, with no complications in its execution.

The RG was performed faster than CG. This result was also reported by other authors^3^. However, in RG, there was a need for greater skill and dexterity in its execution, since the technique requires confidence in releasing the ovarian pedicle. After the removal of the first forcepsthe rotation was slowly undone. Such condition may also be related to the learning curve^8^.

The surgical time was reduced in RG, as it is not necessary to ligate the ovarian pedicles^3^,^4^,^9^. This fact was confirmed in the present study, since the RG applied to the female cats presented shorter surgical time than CG.

The level of difficulty of RG was higher than KG. This may be related to an inadequate essential psychomotor skill to perform the technique *in vivo* 2. This may have resulted in lack of confidence in releasing the ovarian pedicle, and uncertainty in immediate OAVC hemostasis. However, the absence of hemorrhages demonstrated that the technique is safe.

The dexterity acquired during the procedures was fundamental to conduct the techniques satisfactorily. There was no difficulty in exposing the ovarian pedicles. However, OAVC hemostasis was easily observed in KG and RG compared to CG. These were also observed in others studies^2^,^3^.

## Conclusion

Ovarian arteriovenous complex hemostasis techniques without using thread wires or sealing in OH in cats were more efficient, practical and faster than the traditional technique. However, the comparison between the three techniques showed that OAVC rotation resulted in the surgeon’s lack of confidence regarding the effectiveness of OAVC hemostasis.
